# *Limoniastrum monopetalum*–Mediated Nanoparticles and Biomedicines: In Silico Study and Molecular Prediction of Biomolecules

**DOI:** 10.3390/molecules27228014

**Published:** 2022-11-18

**Authors:** Afrah E. Mohammed, Sahar S. Alghamdi, Nada K. Alharbi, Fatma Alshehri, Rasha Saad Suliman, Fahad Al-Dhabaan, Maha Alharbi

**Affiliations:** 1 Department of Biology, College of Science, Princess Nourah bint Abdulrahman University, P.O. Box 84428, Riyadh 11671, Saudi Arabia; 2 Department of Pharmaceutical Sciences, College of Pharmacy, King Saud Bin Abdulaziz University for Health Sciences, Riyadh 11481, Saudi Arabia; 3 King Abdullah International Medical Research Center (KAIMRC), Riyadh 11481, Saudi Arabia; 4 Department of Pharmacy, Fatima College of Health Sciences, Abu Dhabi 3798, United Arab Emirates; 5 Department of Biology, College of Science and Humanities, Shaqra University, Ad-Dawadimi 11911, Saudi Arabia

**Keywords:** silver nanoparticles, antimicrobial, molecular docking, pharmacokinetic prediction, biomolecules

## Abstract

An in silico approach applying computer-simulated models helps enhance biomedicines by sightseeing the pharmacology of potential therapeutics. Currently, an in silico study combined with in vitro assays investigated the antimicrobial ability of *Limoniastrum monopetalum* and silver nanoparticles (AgNPs) fabricated by its aid. AgNPs mediated by *L. monopetalum* were characterized using FTIR, TEM, SEM, and DLS. *L. monopetalum* metabolites were detected by QTOF–LCMS and assessed using an in silico study for pharmacological properties. The antibacterial ability of an *L. monopetalum* extract and AgNPs was investigated. PASS Online predictions and the swissADME web server were used for antibacterial activity and potential molecular target metabolites, respectively. Spherical AgNPs with a 68.79 nm average size diameter were obtained. Twelve biomolecules (ferulic acid, trihydroxy-octadecenoic acid, catechin, pinoresinol, gallic acid, myricetin, 6-hydroxyluteolin, 6,7-dihydroxy-5-methoxy 7-O-β-d-glucopyranoside, methyl gallate, isorhamnetin, chlorogenic acid, 2-(3,4-dihydroxyphenyl)-5,7-dihydroxy-4-oxo-4H-chromen-3-yl 6-O-(6-deoxy-β-l-mannopyranosyl)-β-d-glucopyranoside) were identified. The *L. monopetalum* extract and AgNPs displayed antibacterial effects. The computational study suggested that *L. Monopetalum* metabolites could hold promising antibacterial activity with minimal toxicity and an acceptable pharmaceutical profile. The in silico approach indicated that metabolites 8 and 12 have the highest antibacterial activity, and swissADME web server results suggested the CA II enzyme as a potential molecular target for both metabolites. Novel therapeutic agents could be discovered using in silico molecular target prediction combined with in vitro studies. Among *L. Monopetalum* metabolites, metabolite 12 could serve as a starting point for potential antibacterial treatment for several human bacterial infections.

## 1. Introduction

Antibiotic is the gold standard of infectious disease treatment, though misuse may lead to a variety of undesirable consequences, such as the development of a wide range of resistant bacteria [1]. In relation to the World Health Organization (WHO), MDR is a serious public health risk, as several pathogenic bacteria have evolved as non-sensitive to numerous antibacterial agents [2]. The WHO recently confirmed that new sources of antibiotics are urgently required to reduce the global spread of antibiotic resistance. Different promising approaches have been developed to minimize the wide spread of MDR. Nanotechnology is considering a stepping stone for controlling and preventing the development of MDR. Nanoparticles (NPs) have a variety of biomedical applications, such as antibacterial, antifungal, and antiviral agents. In contrast to bulk materials, NPs have distinct properties, including shape, size, distribution, and surface area, which facilitates their attachment to ligands. The potential of NPs relies mainly on the size of the practical, which improves their ability in bacterial cell penetration, which leads to cell destruction via various mechanisms. Therefore, nano-antibiotics might be considered an excellent substitute to present antimicrobial therapy in combating MDR bacteria. Importantly, they have been approved by the Food and Drug Administration (FDA), and about 1000 commercial nanoproducts are currently available in the market worldwide [3,4]. Besides their well-known effectiveness in the biomedical field, NPs have been widely used in the past few years in various applications, such as electronic, catalysis, and optical applications. Silver nanoparticles (AgNPs) have been widely used due to their high antimicrobial property, which has led to the improvement of a wide variety of silver nanoproducts, such as nano-silver-coated wound dressings [5]. Kovács et al. [6] also evaluated the aptitude of AgNPs for cancer mitigation through different attitudes, such as interaction with cell components, development in tumor tissues besides oxidative stress, and activation of numerous signaling pathways. AgNP syntheses could be established using different techniques, including chemicals and physicals. Producing AgNPs by physicochemical routes requires expensive equipment and long reaction times and releases toxic byproducts that are hazardous to the environment. To conquer these disadvantages, a green synthesis of AgNPs using biogenic agents has become an alternative to chemical and physical synthesis [7]. Recently, green fabrication of nanoparticles utilizing extracts from plants and microorganisms, such as actinomycetes and algae, has drawn great attention due to its simplicity and cost-effectiveness. Extracts from plants are more valuable for several reasons. Plants are easy-to-obtain and low-cost materials [8]. Additionally, plants contain a variety of phytochemicals that can act as Ag ions and Ag atoms, reducing and capping agents, respectively [9]. The synthesized AgNPs by pure phytochemicals or plant extracts have approved several biological activities, such as antifungal, antiviral, antibacterial, and anti-inflammatory effects [10,11]. Varied studies have indicated the ability of plant extracts in metal NP fabrication; for example, El-Zayat et al. [12] reported cytotoxic and antioxidant effects of varied NPs of zinc and selenium prepared using *Ephedra aphylla* extract. Further, garlic and ginger extracts also approved their ability in copper, silver, zinc, and iron NP formulations, which indicated significant antimicrobial activities [13]. 

Currently, we are focusing on *Limoniastrum monopetalum* as a source of active metabolites since it is known as an adapted ground cover to a variety of harsh environmental condition, such as water shortages, high light intensity, and temperatures, and can significantly grow on poor organic soil [14]. *L. monopetalum* is a wild small silvery herb (Plumbaginaceae); it is naturally found in coastal sands and salt marshes along Egypt’s northern coast and other Mediterranean countries. Environmentally, it can stabilize coastal dunes, phytoremediate petroleum hydrocarbon degradation, and reduce heavy metals in polluted areas. Importantly, *L. monopetalum* could also be a potential source of biomolecules as biomedicine [15], since it is tolerant to varied stress conditions. The extracts from *L. monopetalum* displayed variable significant antibacterial potential since they have a great activity against multi-drug-resistant microbes belong to candida species, such as *Candida glabrata* and *C. krusei.* [16]. Recently, we published data regarding biologically active plant metabolites from *Lycium shawii* by an in silico study; such approach could be of interest in searching for biogenic agents as drugs. Biomolecules for a disease suppression process or their exact mode of action could be predicted using an in silico approach [17], which could be of great interest to save resources and time that are needed for in vitro and in vivo studies [18]. Recent in silico molecular docking investigations indicated that the antibacterial mechanism of NPs is the β-lactamase enzyme suppression ability [19,20,21]. Our previous study suggested that emodin, the *L. shawii* metabolites, is targeting the carbonic anhydrase IX enzyme and suppressing its activity [22]. Our investigation used *L. monopetalum*, where the leaves were extracted by water, and the final product has been used as an antibacterial agent and as a mediator in AgNP fabrication. AgNPs were characterized using different approaches, and the metabolites from the plant extract were detected using QTOF–LCMS. Further, in silico molecular target prediction was assessed for the identified biomolecules using different multiple computational approaches in a trial to detect the target of the *L. monopetalum* biomolecules as antibacterial agents.

## 2. Results

In the recent study, NPs were prepared using one biogenic agent, leaf extracts of *L. monopetalum*. The successful fabrication of AgNO_3_ to AgNPs was observed by the color change to dark brown after 6 h when combined with a *L. monopetalum* extract in a time-dependent style. AgNO_3_ was incubated with the *L. monopetalum* extract until no more color alteration was observed, which was as an indication of the total bioconversion of Ag^+^ ions into Ag^0^. A subsequent separation of the biogenic L-AgNPs, 1 mg/mL, was prepared for more analysis. 

### 2.1. Characterizations of the Prepared Nanoparticles

**Figure 1:**
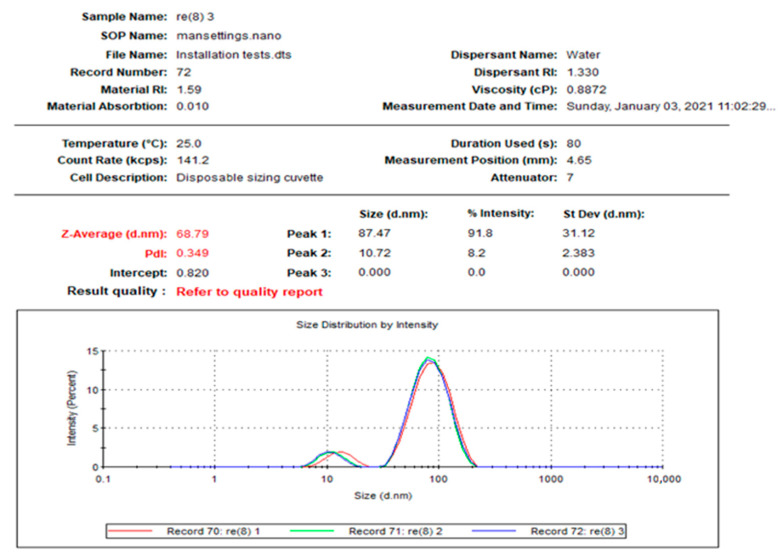
Size distribution of L-AgNPs.

**Figure 2:**
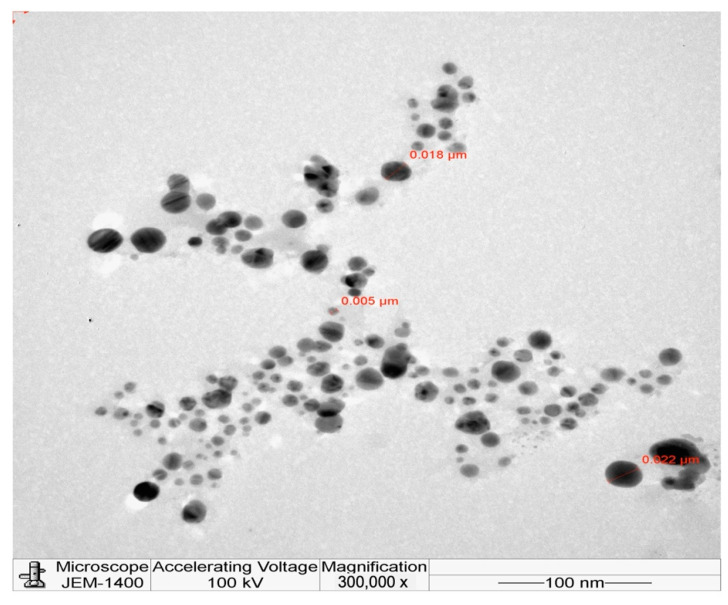
Spherical shape and distribution of L-AgNPs.

**Figure 3:**
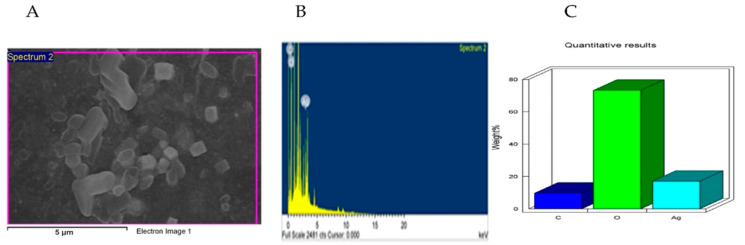
L-AgNP surface morphology (**A**) and quantitative data evaluation of SEM image indicating the weights of the silver, oxygen, and carbon atoms by EDS (**B**,**C**).

**Figure 4:**
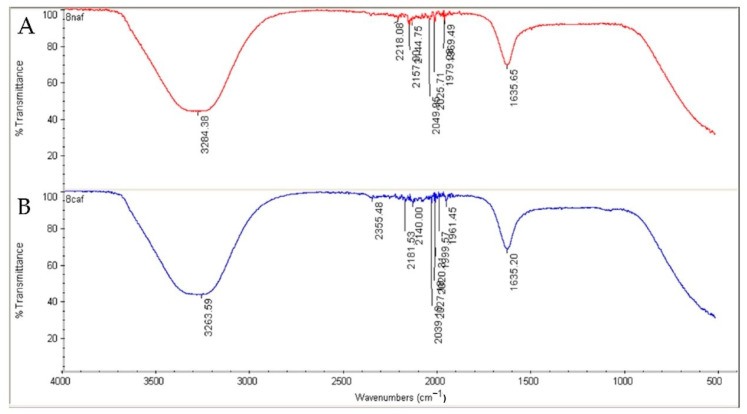
FTIR absorbance peaks of *L*. *monopetalum* NPs (**A**) and *L. monopetalum* extract (**B**) showing the peaks of a functional group of organic material in the extract tested solutions.

Figure 1 reveals the distribution of the L-AgNPs size, indicating a mean size of 68.79 and a 0.349 polydispersity index (PDI). Moreover, L-AgNPs have been investigated by TEM analysis, which illustrates good dispersion, and spherical shapes have been observed, with some NPs indicating size diameters of 5 and 18 nm (Figure 2). A SEM microscope was used for EDX analysis to find out the element composition of the NPs and is presented in Figure 3. Results indicate the presence of carbon, oxygen, and silver at their exact energy values. The morphology of L-AgNPs noted under SEM indicated some spherical nanoforms besides square ones. FTIR is an analytical method used to provide and detect organic and inorganic biomolecules that facilitate the production of NPs from Ag ions. Figure 4 demonstrates the major peaks noted at 3284.38 and 1635.65 cm^−1^ for plant extract and 3263.59 and 1635.20 cm^−1^ for AgNPs in the spectra.

### 2.2. The Antibacterial Activity

**Figure 5:**
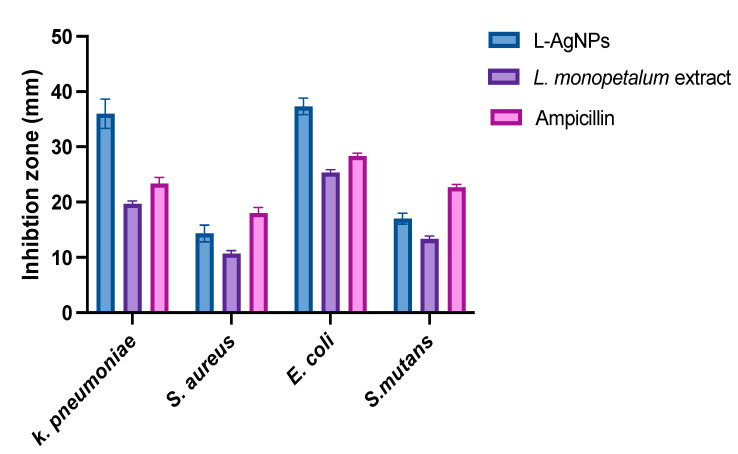
Antimicrobial effect of L-AgNPs, *L. monopetalum* extract, and ampicillin as an inhibition zone (mm).

The antibacterial action of the study agents showed high activity against all tested bacteria, where the inhibition zones of both were greater against Gram-negative *K. pneumoniae* (36 ± 2.6; 19.7 ± 0.5 mm) and *E. coli* (37.3 ± 1.5; 25.3 ± 0.6 mm) for L-AgNPs and a *L. monopetalum* extract, respectively, compared with that against Gram-positive *S. aureus* (14.3 ± 1.5; 10.7 ± 0.6 mm) and *S. mutans* (17.0 ± 1.0; 13.3 ± 0.6 mm) for L-AgNPs and a *L. monopetalum* extract, respectively. Significant differences were noted between the effect of both tested agents against all strains, as presented in Figure 5. Ampicillin was tested as a positive control. Significant variations were noted for the agents tested and bacterial strains, and *p* < 0.0001 was noted also for their interaction.

### 2.3. Identification of the Chemical Components of the Extract

**Figure 6:**
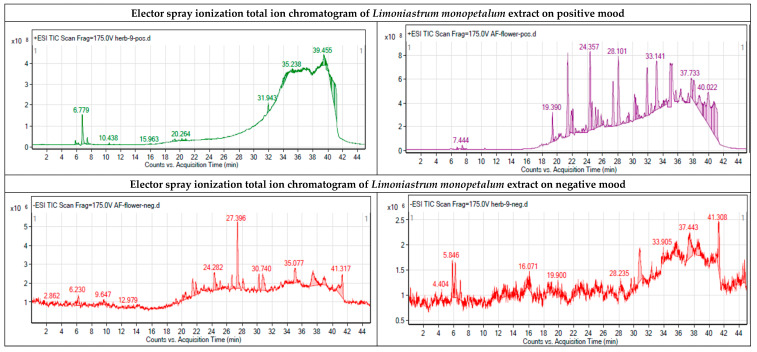

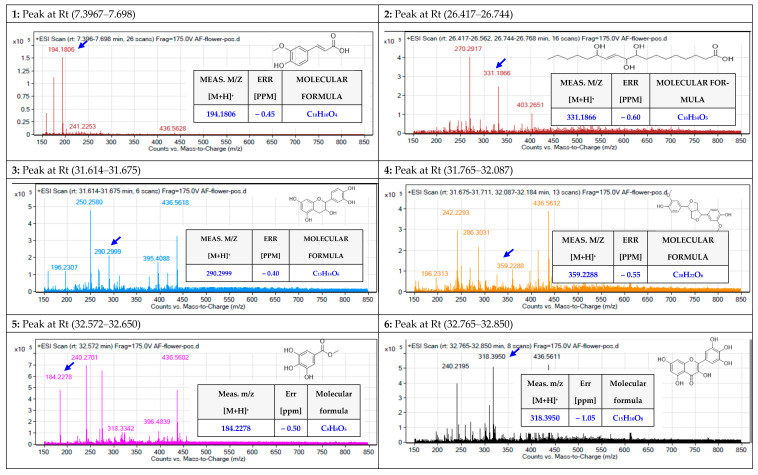

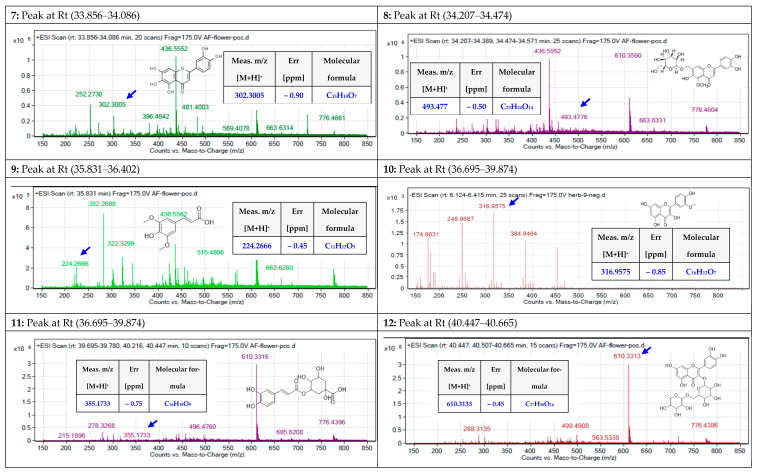
Chromatograms indicating the base peaks of the methanolic extract of *L. monopetalum* and the uncertainly identified biomolecules, which are (1) ferulic acid [23], (2) trihydroxy-octadecenoic acid [24], (3) catechin [24], (4) pinoresinol [24], (5) gallic acid [24], (6) myricetin [24], (7) 6-hydroxyluteolin [24], (8) 6,7-dihydroxy-5-methoxy 7-O-β-d-glucopyranoside [25], (9) methyl gallate [26], (10) isorhamnetin [23], (11) chlorogenic acid [27], and (12) 2-(3,4-dihydroxyphenyl)-5,7-dihydroxy-4-oxo-4H-chromen-3-yl 6-O-(6-deoxy-β-l-mannopyranosyl)-β-d-glucopyranoside, [28]. Mean *m/z* implies measured *m/z*.

Further, total ion current spectra (TIC) raw data for the water extract of the *L*. *monopetalum* is presented in Figure 6, where mass screenings have described the following: detected nodes at different retention times per minute were screened for feature extraction at 6000 counts’ minimum intensity and aligned with earlier reported molecules, taking into account adducts ([M+H]^+^, and [M−H]^−^). The tentatively identified compounds are ferulic acid [23], trihydroxy-octadecenoic acid [24], catechin, [24], pinoresinol [24], gallic acid [24], myricetin [24], 6-hydroxyluteolin [24], 6,7-dihydroxy-5-methoxy 7-O-β-d-glucopyranoside [25], methyl gallate [26], chlorogenic acid [27], isorhamnetin [23], and 2-(3,4-dihydroxyphenyl)-5,7-dihydroxy-4-oxo-4H-chromen-3-yl 6-O-(6-deoxy-β-l-mannopyranosyl)-β-d-glucopyranoside, [28], Mean *m/z* implies measured *m/z*.

### 2.4. In Silico Study

#### 2.4.1. Prediction of Antibacterial Activity

The antibacterial activity of the identified metabolites (Figure 6) was computed using the PASS Online (Way2Drug) web server. As summarized in Table 1, metabolite 12 demonstrated the highest predicted score (0.677), followed by metabolites 8 and 11, which possess moderate predicted activity (0.569 and 0.537, respectively). The activity predicted scores for the remaining metabolites were below 0.5, suggesting that experimental activity is insignificant.

#### 2.4.2. Molecular Target Predictions

**Figure 7:**
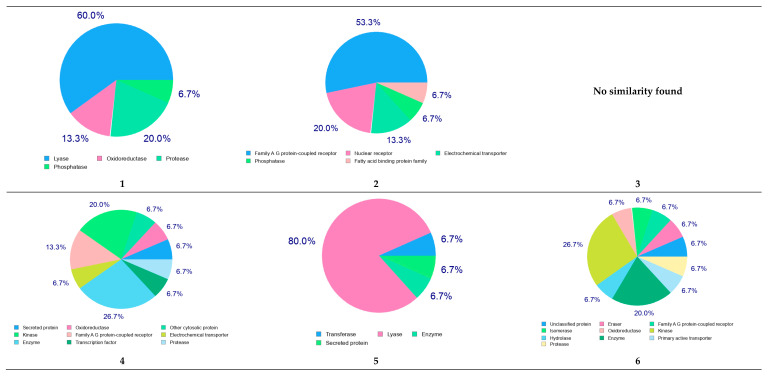

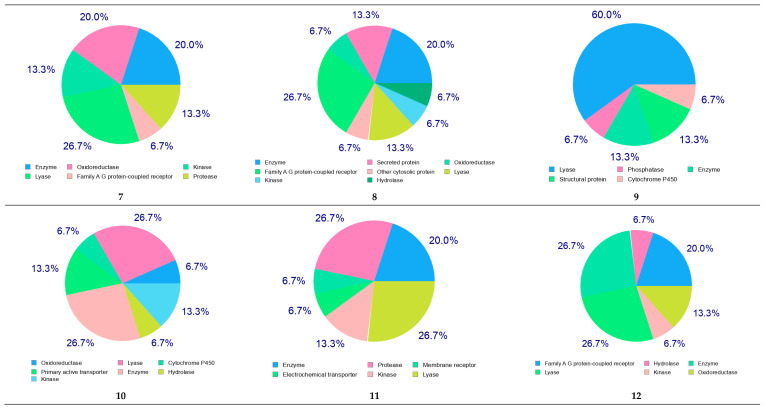
The molecular target prediction for the metabolites using the SwissTargetPrediction web server.

Using the SwissTargetPrediction web server, the potential molecular targets for the identified metabolites were predicted. As shown in Figure 7, metabolite numbers 1, 5, and 7–12 were predicted to act on the lyase enzyme, in particular, carbonic anhydrase II. Additionally, the predicted results suggest that multiple molecular targets could be involved in producing the beneficial antibacterial effect. 

#### 2.4.3. Molecular Docking Study

**Figure 8:**
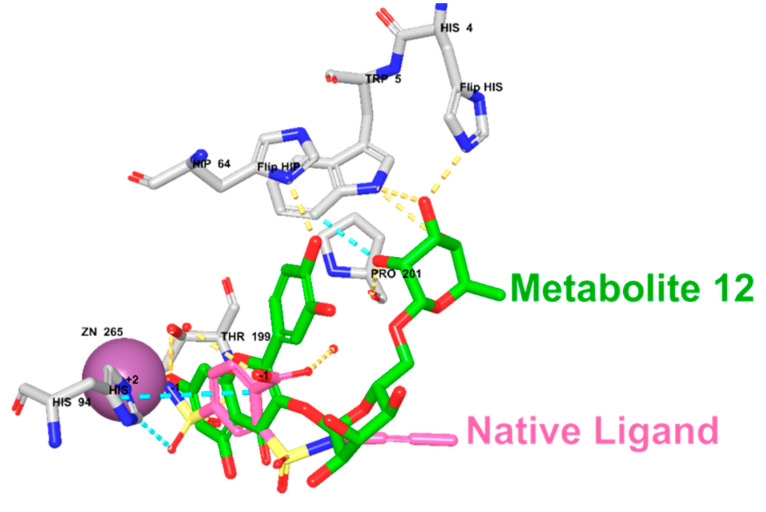

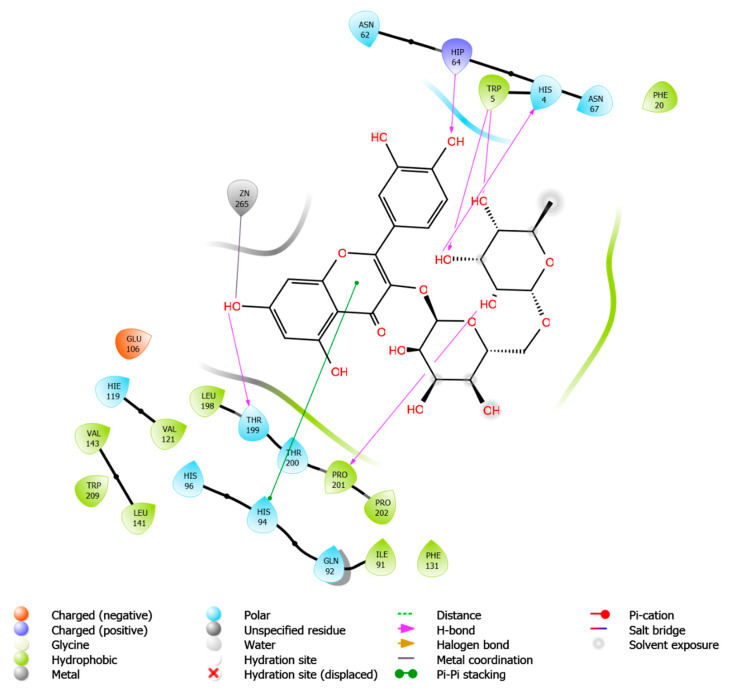
The 3D and 2D molecular interactions of metabolite 12 with the crystal structure of the human carbonic anhydrase II enzyme.

The molecular target prediction results indicated that the carbonic anhydrase II enzyme might be an effective biological target for enhancing the antibacterial potential of the identified metabolites. Thus, to provide additional insights into the molecular interactions of the metabolites with the CA II enzyme, a molecular docking study was conducted using the Maestro software. As summarized in Table 2, metabolite 12 exhibited the highest docking score (−10.37) with several hydrogen bonds and π–π stacking interactions with HIS94, THR199, HIP64, HIS4, TRP5, PRO201, and zinc coordination (Figure 8). Metabolite 8 demonstrated a docking score of −7.89 with the involvement of several amino acid residues, including ASN67, GLU69, THR199, and forming zinc coordination. Our results were consistent with the antibacterial activity prediction results in which metabolites 12 and 8 demonstrated the highest predicted antibacterial activity. It is worth mentioning that the docking score of metabolite 12 was higher than the native ligand, suggesting a potential antibacterial activity for this metabolite.

#### 2.4.4. Pharmacokinetic Parameters Evaluation 

**Figure 9:**
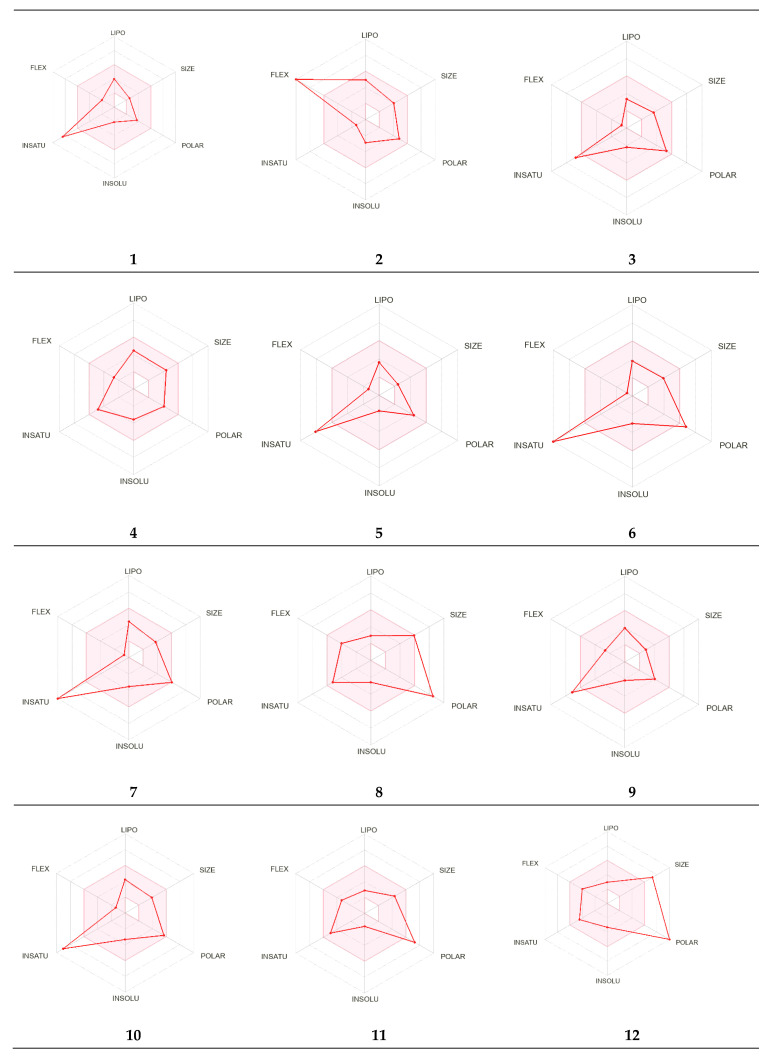
The distribution of the predicted pharmacokinetic characteristics for the detected metabolites. The light red color represents the recommended range for the orally bioavailable drugs, and the dark red line represents the properties of the metabolite. LIPO: lipophilicity; size: molecular weight; POLAR: solubility; INSOLU: insolubility; INSATU: insaturation; and FLEX: flexibility. The properties involved in the colored zone are preferred for orally active drugs.

The evaluated pharmacokinetic properties for the identified metabolites are summarized in Table 3. Based on our results, most of the identified metabolites possessed pharmacokinetic properties that were within the recommended ranges except for metabolites 6, 8, 11, and 12. These four metabolites (6, 8, 11, and 12) violated Lipinski’s rule of 5 on the number of hydrogen bond donors (recommended to be lower than 5) and acceptors (recommended to be less than 10), which could affect the oral absorption (Figure 9). Moreover, metabolite 12 exhibits a molecular weight of greater than 500 daltons, which also violates the acceptable range for orally bioavailable drugs. Additionally, the effects of the metabolites on CYP isoenzymes were assessed, and none of the metabolites were predicted to inhibit CYP2C19 and CYP2C9. Meanwhile, metabolites 6, 7, and 10 were predicted to inhibit CYP1A2; metabolites 2, 4, 7, and 10 inhibit CYP2D6; and metabolites 4, 6, 7, and 10 inhibit CYP3A4. 

#### 2.4.5. Toxicity Assessment

Several organ and endpoint toxicities were assessed and evaluated for the identified metabolites using the ProTox-II web server, including hepatotoxicity, carcinogenicity, immunotoxicity, mutagenicity, and cytotoxicity. As summarized in Table 4, none of the metabolites exhibited hepatotoxicity or cytotoxicity. Meanwhile, metabolites 6 and 7 demonstrated moderate predicted carcinogenicity and mutagenicity. Furthermore, metabolites 1, 2, and 8–12 were predicted to be immunotoxic.

#### 2.4.6. Prediction of Cardiac Toxicity

In addition to the organ and endpoint toxicity, it was crucial to evaluate the blockage of the hERG channel by metabolites, which could potentially lead to cardiac toxicity. Thus, the metabolites were assessed for cardiac toxicity using a pred-hERG web server. Moreover, the probability map demonstrates the involvement of the atoms in the cardiotoxicity as the green color increases with more contour lines representing greater involvement and contribution of the atoms (Table 5, probability map). As summarized in Table 5, metabolites 3, 4, 8, and 12 were predicted to be potentially cardiotoxic with confidences of 50%, 50%, 50%, and 60%, respectively. Meanwhile the remaining metabolites were predicted to be noncardiotoxic. 

## 3. Discussion

The study validates the formation of L-AgNPs using leaf extracts of *L. monopetalum*. The L-AgNPs were successfully synthesized and observed initially by changing the visible color of the mixture of a *L. monopetalum* extract and AgNO_3_ to dark brown color after 6 h from mixing of the two components. The obtained result was as an indication of the entire biotransformation and conversion of Ag^+^ ions into Ag^0^, and the brown color is a distinctive feature of the generation of plasmon vibration excitation on the surface of L-AgNPs [29]. Further, the formation of L-AgNPs was verified by a dynamic light scattering (DLS) system to demonstrate the size distribution. The size distribution provides evidence of L-AgNP production with a mean size of 68.79 nm with a 0.349 polydispersity index (PDI). The size and density of the produced L-AgNPs suggest a lack of formation of Ag clusters or ultrasmall AgNPs [30].

The obtained L-AgNPs are further corroborated by TEM. A representative TEM image of the L-AgNPs indicates their varied size diameters of 5 and 18 nm and shows the spherical shapes of almost all AgNPs. The size of the NPs that is detected using TEM is normally small compared with that noted by DLS, which is reported in our findings. Such variation could be related to the fact that both techniques were varied in their principles, where DLS generally assesses the NPs’ hydrodynamic radius [31]. Besides that, the amount of the sample is much higher than that used for TEM analysis; therefore, impurities of phytochemicals that cap the NPs could also be present. Compared with other studies, the obtained TEM image also shows most of the AgNPs without aggregation as much individual particles were seen [32,33]. The absence of aggregation might be suggested by NPs’ surface capping by the *L. monopetalum* molecules. These capping agents are also crucial to enhance NPs’ biomedical functionality. The elemental assessment of NPs was conducted by SEM using EDX analysis, which approves the occurrence of carbon, oxygen, and silver atoms. The peaks detected around 3.0 keV are characteristic of metallic silver in the generated NPs. The presence of carbon and oxygen is due to the molecules of the used *L. monopetalum* leaf extracts. The biomolecules that are possibly found in a *L. monopetalum* extract could be responsible for the easy fabrication of AgNO_3_ to AgNPs [34] and can be detected by the FTIR technique. Such molecules could also be accountable for the extract’s biological activity. Our findings demonstrated major peaks at 1635.65 and 1635.20 cm^−1^, which can be ascribed to amide I for the carbonyl stretch in proteins [35]. The peaks at 3284.38 and 3263.59 could be assigned for polyphenolic and protein peptide [36]. The occurrence of several functional groups in an NP solution suggests their role in the fabrication process. The peaks from both tested agents showed slight variation in the absorption band magnitude, which might suggest the utilization of plant metabolites in NPs fabrication. The antimicrobial effects of the L-AgNPs and *L. monopetalum* extract was examined against two Gram-positive bacteria (*S. aureus* and *S. mutans*) and two Gram-negative bacteria (*K. pneumonia* and *E. coli*). The result confirms the effectiveness of both the L-AgNPs and *L. monopetalum* extract on all tested bacteria strains. However, the inhibition effect by the obtained L-AgNPs on all selected bacteria was greater than that of the *L. monopetalum* extract. Moreover, both L-AgNPs and the *L. monopetalum* extract exhibited excellent growth inhibition against Gram-negative bacteria more than that observed for Gram-positive bacteria, which might suggest better penetration ability of the NPs in the cell of Gram-negative bacteria due to the natural barrier made of a thinner peptidoglycan layer compared with that found in the cell of Gram-positive bacteria [37]; consequently, a high effect was noted. Previous studies showed that a *L. monopetalum* leaf extract alone has a high level of phenolic and flavonoid compounds [13,23], and the phenolic compounds (phenolic hydroxyl groups) significantly increase the antibacterial potential by inhibiting microbial enzymes and increasing affinity to cytoplasmic membranes [38]. The strong antibacterial properties of the obtained L-AgNPs are properly due to their size and biomolecule attached over their surface [39]. Another study by Martínez-Castañon et al. stated that the smaller AgNP size, the grater their antibacterial activity [40]. *E. coli* shows the highest inhibition zone among the four tested bacteria (37.3 ± 1.5 mm) by L-AgNPs, which is not too far from that of the *K. pneumoniae* (36 ± 2.6 mm). The antimicrobial results of the L-AgNPs were in agreement with other studies that used green synthesis AgNPs [11,41,42]. Not only the NPs’ size affects the antibacterial properties, but also the capping agent enhances antibacterial action [39]. Damage of bacterial cell membranes was suggested by several studies as AgNPs can cause structural changes by adhering to the negative charge lipopolysaccharides (LPS), which disrupt membrane permeability and lead to bacterial death [43,44]. Further, a study demonstrates that the alteration of the membrane in *K. pneumoniae* and *E. coli* was proposed as a mode of action of AgNPs [45]. In addition, in a report by Gopinath et al., SEM images indicated a damaged bacterial membrane after treatment with AgNPs [46]. Altering membrane functionality is the target of many antibacterial drugs. In contrast, the use of L-AgNPs is environmentally safe approach, cost-effective and overcomes the side effects associated with these antibacterial drugs. Possible antimicrobial actions for the biofabricated AgNPs might be linked to their biocompatibility, increasing the ROS production and hurting a bacterial cell [47,48].

The activity of the plant extract as an antimicrobial agent or biomediator in NP fabrication could mainly rely on its active ingredients that have been detected using QTOF–LCMS. Twelve components appeared as 12 peaks were identified from a *L. monopetalum* extract where: Peak 1: the value of *m/z* at 7.396–7.698 (retention time) has been linked to the parent compound ferulic acid [23], with the molecular formula of [C_10_H_10_O_4_]^+^ and *m/z* [M+H]^+^ 194.0812 daltons, in the positive ion mode [M+H]^+^ *m/z* with 195.1806 daltons and the negative mode of [M−H]^−^ with *m/z* 193.806 daltons, demonstrating 194.18 g mol^−1^ as the molecular weight of the compound. Peak 2: the value of *m/z* at 26.417–26.744 (retention time) has been linked to the parent compound trihydroxy-octadecenoic acid [24], with the x molecular formula of [C_18_H_34_O_5_]^+^ and *m/z* [M+H]^+^ of 331.1866 daltons, in the positive ion mode [M+H]^+^ *m/z* with 330.24 daltons and the negative mode [M-H]^-^ with *m/z* 229 daltons, demonstrating 330.460 g mol^−1^ as the molecular weight of the compound. Peak 3: the value of *m/z* at 31.614–31.675 (retention time) has been linked to the parent compound catechin [24], with the molecular formula of [C_20_H_22_O_6_]^+^ and *m/z* [M+H]^+^ 360.2078 daltons, in the positive ion mode and the negative mode [M−H]^−^ with *m/z* 358.087 daltons, demonstrating 359.23 g mol^−1^ as the molecular weight of the compound. Peak 4: the value of *m/z* at 31.765–32.087 (retention time) has been linked to the parent compound pinoresinol [24], with the molecular formula of [C_18_H_34_O_5_]^+^ and *m/z* [M+H]^+^ 331.1866 daltons, in the positive ion mode [M+H]^+^ with *m/z* 330.24 daltons and the negative mode [M−H]^−^ with *m/z* 229 daltons, demonstrating 330.460 g mol^−1^ as the molecular weight of the compound. Peak 5: the value of *m/z* at 32.572 (retention time) has been linked to the parent compound gallic acid [24], with the molecular formula of [C_10_H_10_O_4_]^+^ and *m/z* [M+H]^+^ 184.2278 daltons, in the positive ion mode [M+H]^+^ with *m/z* 184.15 daltons and the negative mode [M−H]^−^ with *m/z* 183.806 daltons, demonstrating 184.18 g mol^−1^ as the molecular weight of the compound. Peak 6: the value of *m/z* at 32.765–32.850 (retention time) has been linked to the parent compound myricetin [24], with the molecular formula of [C_15_H_10_O_8_]^+^ and *m/z* [M+H]^+^ 318.3950 daltons, in the positive ion mode of [M+H]^+^ with *m/z* 318.24 daltons and the negative mode [M−H]^−^ with *m/z* 317.023 daltons, demonstrating 318.26 g mol^−1^ as the molecular weight of the compound. Peak 7: the value of *m/z* at 33.856–34.086 (retention time) has been linked to the parent compound 6-hydroxyluteolin [24], with the molecular formula of [C_15_H_10_O_7_]^+^ and *m/z* [M+H]^+^ 302.236 daltons, in the positive ion mode of [M+H]^+^ with *m/z* 302.826 daltons and the negative mode [M−H]^−^ with *m/z* 300.261 daltons, demonstrating that the compound has a molecular weight of 302.4 g mol^−1^. Peak 8: the value of *m/z* at 34.207–34.474 (retention time) has been linked to the parent compound 6,7-dihydroxy-5-methoxy 7-O-β-d-glucopyranoside [25], with the molecular formula of [C_23_H_24_O_12_]^+^ and *m/z* [M+H]^+^ 493.477 daltons, in the positive ion mode [M+H]^+^ with *m/z* 492.43 daltons and the negative mode [M−H]^−^ with *m/z* 491.35 daltons, demonstrating 492.24 g mol^−1^ as the molecular weight of the compound. Peak 9: the value of *m/z* at 35.831–36.402 (retention time) has been linked to the parent compound methyl gallate [26], with the molecular formula of [C_11_H_12_O_5_]^+^ and *m/z* [M+H]^+^ 224.210 daltons, in the positive ion mode [M+H]^+^ with *m/z* 224.2666 daltons and the negative mode of [M−H]^−^ with *m/z* 223.038 daltons, demonstrating 224.210 g mol^−1^ as the molecular weight of the compound. Peak 10: the value of *m/z* at 36.147–36.465 (retention time) has been linked to the parent compound isorhamnetin [23], with the molecular formula of [C_16_H_12_O_7_]^+^ and *m/z* [M+H]^+^ 316.9585 daltons, in the positive ion mode [M+H]^+^ with *m/z* 317.26 daltons and the negative mode [M−H]^−^ with *m/z* 315.73 daltons, demonstrating 316.69 g mol^−1^ as the molecular weight of the compound. Peak 11: the value of *m/z* at 36.695–39.874 (retention time) has been linked to the parent compound chlorogenic acid [27], with the molecular formula of [C_16_H_18_O_9_]^+^ and *m/z* [M+H]^+^ 355.1733 daltons, in the positive ion mode [M+H]^+^ with *m/z* 354.10 daltons and the negative mode [M-H]^-^ at *m/z* 353.28 daltons, demonstrating 354.10 g mol^−1^ as the molecular weight of the compound. Peak 12: the value of *m/z* at 40.447–40.665 (retention time) has been linked to the parent compound 2-(3,4-dihydroxyphenyl)-5,7-dihydroxy-4-oxo-4H-chromen-3-yl 6-O-(6-deoxy-β-l-mannopyranosyl)-β-d-glucopyranoside [28], with the molecular formula of [C_27_H_30_O_16_]^+^ and *m/z* [M+H]^+^ 610.3313 daltons, in the positive ion mode of [M+H]^+^ with *m/z* 610.15 daltons and the negative mode [M-H]^-^ with *m/z* 609.780 daltons, demonstrating 610.52 g mol^−1^ as the molecular weight of the compound. Further, in this study, we examined the application of a *L. Monopetalum* extract as a potential antibacterial therapy by using multiple computational approaches to predict the antibacterial activity of the identified metabolites, their possible molecular targets, active site interactions, pharmacokinetic properties, and toxicity. According to PASS Online predictions, metabolite 12 (2-(3,4-dihydroxyphenyl)-5,7-dihydroxy-4-oxo-4H-chromen-3-yl 6-O-(6-deoxy-β-l-mannopyranosyl)-β-d-glucopyranoside) and metabolite 8 (6,7-dihydroxy-5-methoxy 7-O-β-d-glucopyranoside) demonstrated the highest antibacterial activity, and SwissADME web server results suggested the CA II enzyme as a potential molecular target for both metabolites with other targets that could be involved. Since several studies have reported that inhibitors of carbonic anhydrase enzymes could serve as a novel approach for new classes of antibacterial agents [49,50,51], the metabolites were docked into the crystal structure of human carbonic anhydrase II. Interestingly, metabolite 12 demonstrated the highest docking score, followed by metabolite 8, which both were aligned with our antibacterial activity predictions. Moreover, computational pharmacokinetic prediction results suggested that metabolite 12 violated the ROF that could affect the route of the administration, while metabolite 8 was within the recommended range for oral bioavailability. Additionally, metabolites 8 and 12 demonstrated no inhibition effect on five CYP isoenzymes, suggesting a lower tendency for drug–herbal interaction. Regarding the safety studies, the ProTox-II web server predicted that metabolites 8 and 12 could possess a potential immunotoxicity, while the pred-hERG web server predicted that these two metabolites could exhibit weak to moderate cardiac toxicity. Moreover, additional in vitro and in vivo studies need to confirm the predicted results for the metabolites. Nonetheless, the results of the computational work suggest that metabolite 12 could serve as a starting point for a potential antibacterial treatment for several human bacterial infections.

## 4. Materials and Methods

### 4.1. Plant Materials and Morphological Identification

Leaves were collected from 6-week-old *Limoniastrum monopetalum* obtained from the nursery of the Royal Commission for Riyadh City (RCRC), Riyadh, Saudi Arabia, in January 2021. Plants were characterized and confirmed at Princess Nourah bint Abdulrahman University (Riyadh, Saudi Arabia). Distilled water was used for the cleaning of plant leaves, which were then dried at room temperature and milled by a milling machine (IKA Werke GMBH and Co., Staufen im Breisgau, Germany) to attain a fine powder. The consequent material was kept in a plastic container for further analysis at room temperature.

### 4.2. Preparation of Limoniastrum Monopetalum Extract

An aqueous extract (2% *w*/*v*) of *L. monopetalum* leaves was made by adding 2 g of plant powder to 100 mL distilled water and heated at 90 °C for 15 min using water path. Then the mixture was filtered by filter paper (Whatman No. 1), which was used immediately for NP fabrication.

### 4.3. Preparation of AgNPs 

An amount of 10 mL of an *L. monopetalum* extract and 90 mL of (1 mM) AgNO_3_ were mixed, thereafter heated at 90 °C for 10 min. The mixture was maintained at room temperature in dark conditions. The color alteration was reported every 2 h while waiting for a final solution with a stable brown dark color, which indicated the NPs’ formation by *L. monopetalum* (L-AgNPs). Subsequently, the resultant mixture was centrifuged for 30 min at 13,000 rpm, followed by washing the pellet two times using distilled water; then at the same conditions, the centrifugation process was repeated. Further, the pellet was preserved for drying at room temperature. Following the accomplishment of the AgNP synthesis, a NP concentration of 1 mg/mL was taken for additional use.

### 4.4. Characterization of L-AgNPs 

L-AgNPs were characterized by various approaches applying hydrodynamic size evaluation, which was carried out using Nano ZSP Zetasizer by a dynamic light scattering (DLS) system (MAL1034318, ver 7.11, Serial Number, Malvern Instruments Ltd., Malvern, UK). The L-AgNPs structure and size distribution were carried out by transmission electron microscopy (TEM) by a TEM system (JEM-1011, Jeol, Tokyo, Japan) at an 80 kV voltage. For the detection of the presence of the element silver in the synthesized NPs and for investigating the surface and size of NPs, energy dispersive X-ray spectroscopy (EDS) was performed, applying a scanning electron microscope (SEM) (Jeol JED-2200 series).

### 4.5. Analysis of Surface Functional Groups 

To check the capping agent from the plant extracts adsorbed on the surface of the NPs, Fourier-transform infrared spectroscopy (FTIR) analysis was taken, applying an FTIR spectrometer (Spectrum 100, PerkinElmer, Wellesley, MA, USA), at wavenumber range of 450–3500 cm^−1^ measured in diffuse reflectance.

### 4.6. Antibacterial Screening

The *L. monopetalum* extract and biogenic L-AgNPs were evaluated as antibacterial agents against four bacterium types: methicillin-resistant *Staphylococcus aureus* (MRSA), *Escherichia coli, Streptococcus mutans*, and *Klebsiella pneumoniae*, which were obtained from the Bio House Medical Lab in Riyadh, Saudi Arabia. The well diffusion assay was applied on the selected strain to detect the activity of the tested agents. Bacterial suspensions 0.5 McFarland concentration at 1.5 × 10^8^ CFU/mL were made in saline by the straight colony suspension methodology. The selected inoculums were individually spread on the entire agar surface of the agar plates. Subsequently, holes (0.4 mm diameter each) were made and 40 µL of 1 mg/mL of L-AgNP solution was poured into the well. Additionally, a *L. monopetalum* extract was also tested, and ampicillin was used as a positive control. Before incubation, plates were set aside to dry under aseptic conditions for 1 h and the kept at 37 °C for 24 h. After that, the diameters of the inhibition growth area were assessed in mm. Distilled water was used as a negative control in this study.

### 4.7. LC–QTOF–MS Analysis for Metabolites Detection 

The powder from *L. monopetalum* was soaked in distilled water for 48 h at 60 °C for sample preparation to LC–QTOF–MS analysis for metabolite detection. Filter paper, Whatman Grade No. 1, was used for mixture filtration, then evaporated. Methanol (1 mL) was used for dissolving the aqueous extract (1 mg). An Agilent Extend-C18 column (2.1 mm × 50 mm, 1.8 μm) was used for the separation process aided with elution gradient 0–1 min, 5% B; 1–11 min, 5%–100% B; 11–13 min, 95% B; 13–15 min, 5% B; 15–16 min, 5% B, applying 0.1% HCOOH in water (mobile phase A and 0.1% HCOOH in methanol (mobile phase B), and 10 μL and 300 μL/min were the injection volume and sample flow rate, respectively. The MS1 acquisition approach was obtained using positive mode 100 to 600 *m/z* as mass range. Conditions of the mass spectrometer were designed at 300 °C = gas temperature; 8 I/min = gas flow; 35 psig = nebulizer; 350 °C = sheath gas temperature, and 11. MS1 = sheath gas flow. Data were obtained by a quantitative and qualitative analysis software (Agilent MassHunter, Agilent Technologies). A mass assessment of the spectrum was detected, and LC-MS data were used for extracting the chemical features by the recursive analysis workflow and molecular feature extraction (MFE) algorithm [22].

### 4.8. Antibacterial Activity Prediction

Each identified metabolite was assessed for the antibacterial activity using the online web server Prediction of Activity Spectra for Substances (PASS) (http://www.pharmaexpert.ru/passonline, accessed on 20 June 2022). The web server predicts the activity of small molecules based on the database, and the results are represented by the probability of the compound possessing activity (P_a_) or inactivity (P_i_) at a variety of pharmacological activities. The small molecule is predicted to be highly active experimentally if P_a_ is greater than 0.7, while the range of 0.7 to 0.5 predicts moderate activity, and lower than 0.5, the biological effect is negligible [52].

### 4.9. Target and Pharmacokinetic Prediction

Molecular target predictions and multiple pharmacokinetic parameters were computed by applying the SwissADME web server (http://www.swissadme.ch/index.php, accessed on 20 June 2022) [53]. The Simplified Molecular Input Line Entry System (SMILES) of each metabolite was used as an input to generate these predictions. Several pharmacokinetic properties were computed, including physicochemical parameters, lipophilicity, absorption, distribution, metabolism, and druglikeness according to Lipinski’s rule of five (ROF) [54,55].

### 4.10. Molecular Docking Study

Molecular docking for identified metabolites was performed using the Maestro Schrödinger software (Schrödinger, New York, NY, USA). The crystal structure of human carbonic anhydrase II (hCA II, PDB ID: 5AML, resolution 1.36 Å) was prepared using the protein preparation wizard tool in Maestro. The chemical structures were arranged by the LigPrep tool, and the grid of the active site was created using the receptor grid generation tool in Maestro. The docking results were subjected to quantitative (docking scores ranking) and qualitative (molecular interactions involved) analysis.

### 4.11. Organ and Endpoint Toxicity Assessment 

The freely available web server ProTox-II was utilized to predict the toxicity of each metabolite (https://tox-new.charite.de/protox_II/, accessed on 21 June 2022). Several toxicity endpoints were evaluated, including hepatotoxicity, carcinogenicity, immunotoxicity, mutagenicity, and cytotoxicity [56].

### 4.12. Cardiac Toxicity Prediction

The blockage of hERG K(+) channels can lead to fatal cardiac arrhythmia and toxicity; thus, the metabolites were assessed using the pred-hERG 4.2 web server (http://predherg.labmol.com.br, accessed on 21 June 2022). The results are presented as probability prediction (cardiotoxic versus noncardiotoxic) with confidence and probability maps to visualize the contribution of each atom to the predicted toxicity. The green color (the darker the color with more lines suggests higher involvement in the cardiac toxicity) in the probability map indicates the involvement of the atom in the blockage of hERG, while the pink color indicates the contribution to lowering the cardiac toxicity, and the gray color indicates no involvement in the toxicity [57].

### 4.13. Statistical Analysis

All assessments were carried out in triplicate. Data were presented as mean ± standard deviation (SD). Differences among the investigated components were statistically gained by two-way analysis of variance (ANOVA) by the Prism 9.1 software (GraphPad Software Inc., La Jolla, CA, USA).

## 5. Conclusions

The aqueous extract of *L. Monopetalum* revealed an ability in AgNP fabrication, and the in vitro antibacterial effect was approved for both agents. The inhibitory action of the plant extract and AgNPs was higher against Gram-negative bacteria than that against Gram-positive-tested bacteria. Twelve biomolecules were identified in the *L. Monopetalum* extract by QTOF–LCMS analysis, and our computational results suggested that *L. Monopetalum* metabolites could hold promising antibacterial activity with minimal toxicity and an acceptable pharmaceutical profile. Additional experimental work needs to confirm the computed antibacterial, ADME, and toxicity predictions for the identified metabolites. Moreover, isolation, in vitro, and in vivo antibacterial assessment for each metabolite present in a *L. Monopetalum* extract could be the future direction to identifying new antibacterial agents from natural products.

## Figures and Tables

**Table 1:** The computed antibacterial activity for the identified metabolites.

Biological Activities for Metabolites (Antibacterial)	P_a_	P_i_
1	0.333	0.048
2	0.388	0.033
3	0.320	0.053
4	0.274	0.070
5	0.349	0.043
6	0.421	0.025
7	0.395	0.031
8	0.569	0.011
9	0.359	0.041
10	0.375	0.037
11	0.537	0.013
12	0.677	0.005

**Table 2:** The docking scores of the metabolites with the CA II enzyme.

Metabolite Number	Glide Docking Score	Molecular Interactions
1	−4.65	THR199 and zinc coordination
2	−4.97	ASN67, THR200, PRO201, and zinc coordination
3	−5.347	HIS94, THR200, and zinc coordination
4	−5.16	ASN62, ASN67, THR199, and zinc coordination
5	−4.88	THR200 and zinc coordination
6	−6.12	GLU92, THR199, and zinc coordination
7	−5.85	HIP64, THR199, and zinc coordination
8	−7.89	ASN67, GLU69, THR199, and zinc coordination
9	−5.18	ASN67, THR199, and zinc coordination
10	−4.64	GLN92
11	−6.40	ASN67, GLU69, THR199, THR200, and zinc coordination
12	−10.37	HIS94, THR199, HIP64, HIS4, TRP5, PRO201, and zinc coordination
51J (Native ligand)	−9.580	ASN67, GLN92, THR199, THR200, and zinc coordination

**Table 3:** Pharmacokinetic properties for the identified metabolites using the SwissADME web server.

Properties	Parameters	1	2	3	4	5	6	7	8	9	10	11	12
Physicochemical Properties	MW (g/mol)	194.18	330.46	290.27	358.39	184.15	318.24	302.24	492.43	224.21	316.26	354.31	610.52
	HBA	4	5	6	6	5	8	7	12	5	7	9	16
	HBD	2	4	5	2	3	6	5	7	2	4	6	10
Lipophilicity Log Po/w	iLOGP	1.62	3.18	1.47	2.67	0.97	1.08	1.94	2.00	1.63	2.35	0.96	1.58
	XLOGP3	1.51	3.15	0.36	2.28	0.86	1.18	2.17	−0.39	1.46	1.87	−0.42	−0.33
	MLOGP	1.00	2.01	0.24	1.17	0.18	−1.08	−0.56	−2.43	0.73	−0.31	−1.05	−3.89
Absorption	Water solubility	Soluble	Soluble	Soluble	Moderately soluble	Soluble	Soluble	Soluble	Soluble	Soluble	Soluble	Soluble	Soluble
	GI	High	High	High	High	High	Low	High	Low	High	High	Low	Low
	Log Kp (skin permeation) cm/s	−6.41	−6.08	−7.82	−6.87	−6.81	−7.40	−6.60	−9.58	−6.63	−6.90	−8.76	−10.26
Distribution	BBB permeant	Yes	No	No	Yes	No	No	No	No	No	No	No	No
Metabolism	CYP1A2 inhibitor	No	No	No	No	No	Yes	Yes	No	No	Yes	No	No
	CYP2C19 inhibitor	No	No	No	No	No	No	No	No	No	No	No	No
	CYP2C9 inhibitor	No	No	No	No	No	No	No	No	No	No	No	No
	CYP2D6 inhibitor	No	Yes	No	Yes	No	No	Yes	No	No	Yes	No	No
	CYP3A4 inhibitor	No	No	No	Yes	No	Yes	Yes	No	No	Yes	No	No
Druglikeness	Lipinski	Yes; 0 violation	Yes; 0 violation	Yes; 0 violation	Yes; 0 violation	Yes; 0 violation	Yes; 1 violation: NHorOH > 5	Yes; 0 violation	No; 2 violations: NorO > 10, NHorOH > 5	Yes; 0 violation	Yes; 0 violation	Yes; 1 violation: NHorOH > 5	No; 3 violations: MW > 500, NorO > 10, NHorOH > 5

**Table 4:** Toxicity evaluation for the identified metabolites using the ProTox-II web server.

Metabolite Number	Classification				
	Organ Toxicity (%Probability)	Toxicity Endpoint (% Probability)			
	Hepatotoxicity	Carcinogenicity	Immunotoxicity	Mutagenicity	Cytotoxicity
1	Inactive (0.51)	Inactive (0.61)	Active (0.91)	Inactive (0.96)	Inactive (0.88)
2	Inactive (0.74)	Inactive (0.55)	Inactive (0.99)	Inactive (0.95)	Inactive (0.58)
3	Inactive (0.72)	Inactive (0.51)	Inactive (0.96)	Inactive (0.55)	Inactive (0.84)
4	Inactive (0.86)	Inactive (0.51)	Active (0.89)	Inactive (0.77)	Inactive (0.98)
5	Inactive (0.62)	Inactive (0.63)	Inactive (0.98)	Inactive (0.91)	Inactive (0.93)
6	Inactive (0.69)	Active (0.68)	Inactive (0.86)	Active (0.51)	Inactive (0.99)
7	Inactive (0.69)	Active (0.68)	Inactive (0.97)	Active (0.51)	Inactive (0.99)
8	Inactive (0.85)	Inactive (0.9)	Active (0.98)	Inactive (0.69)	Inactive (0.55)
9	Inactive (0.54)	Inactive (0.67)	Active (0.89)	Inactive (0.87)	Inactive (0.96)
10	Inactive (0.72)	Inactive (0.68)	Active (0.58)	Inactive (0.94)	Inactive (0.95)
11	Inactive (0.72)	Inactive (0.68)	Active (0.99)	Inactive (0.93)	Inactive (0.80)
12	Inactive (0.80)	Inactive (0.91)	Active (0.98)	Inactive (0.88)	Inactive (0.64)

**Table 5:** Cardiac toxicity of the identified metabolites using the pred-hERG web server.

Metabolite Number	Prediction/Potency	Confidence	Probability Map
1	Noncardiotoxic (−)	80%	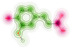
2	Noncardiotoxic (−)	60%	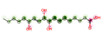
3	Potentially cardiotoxic (+)	50%	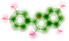
4	Potentially cardiotoxic (+)	50%	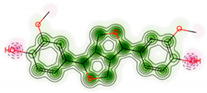
5	Noncardiotoxic (−)	80%	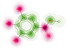
6	Noncardiotoxic (−)	60%	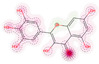
7	Noncardiotoxic (−)	50%	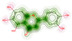
8	Potentially cardiotoxic (+)	50%	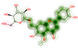
9	Noncardiotoxic (−)	80%	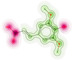
10	Noncardiotoxic (−)	60%	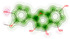
11	Noncardiotoxic (−)	50%	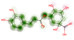
12	Potentially cardiotoxic (+)	60%	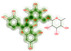

## Data Availability

Data will be available upon request to the corresponding author.

## Sample Availability

Samples of the materials tested are available from the authors.
